# Supplementary material for: Metabolism of Terephthalic Acid by a Novel Bacterial Consortium Produces Valuable By-Products
Source: Microorganisms. 2025 Sep 6;13(9):2082. doi: 10.3390/microorganisms13092082 (PMC12472019; doi:10.3390/microorganisms13092082)
Supplement: Supplementary file 1 [file microorganisms-13-02082-s001.zip › microorganisms-3837895-supplementary.pdf]

Supplemental Materials

Gene Name	Genome ID [Gene location]	Sequence (5' -> 3')	PCR Product Size (bp)	Annealing Temp. (℃)
rpoA	NZ_CP010026.1: 2025942-2026919	GATCCTGCTGCGTCCGGT	95	60
		GGATCAGGTCGCCGATGTAG		
rpoB	NZ_CP010026.1: 1997473-2001579	AACTCGCTCGCTCTGTACG	128	60
		ACGTAACGGCCTTCTTCGAT		
Cpn60	NZ_CP010026.1: 105856-107496	AACCCGTTCGTGCTGCTG	72	60
		GACCTGTTCCAGAATCGGCA		
tphA2I	CP099647.1: 3849026-3850279	GCGCTCGTTCCATGAAGGTA	146	60
		GCCGCACACCCAAAGTATTC		
tphBI	CP099645.1: 3048333-3049280	TCCTGAAATCGCGGTCAAGG	149	60
		TGAGTTCGGGACAACACTGG		
catA	NZ_CP010026.1: c4160839-4159931	CCGTATCTGTGGGACGACTT	139	60
		TGAACAGCGTGAAGTCGAAA		
pcaB	NZ_CP010027.1: 2656776-2658155	GGCCGCACCTGGCTG	124	60
		GCAGCACCAGCACGC		
pcaC	NZ_CP010027.1: 2658949-2659344	TGGGGCGAAATCTGGACG	80	60
		TTGAGCGCGACCATCATC		
pcaD	NZ_CP010027.1: 2658161-2658952	GCGAATTTCTGCGGTCTGTC	75	60
		TTCGAGACGATTCCCGTGAC		

Supplemental Table S1: RT-qPCR Primer Sequences.
Accession numbers for genes of interest were identified from NCBI database. Forward and reverse primer sequences were designed using Primer-BLAST.

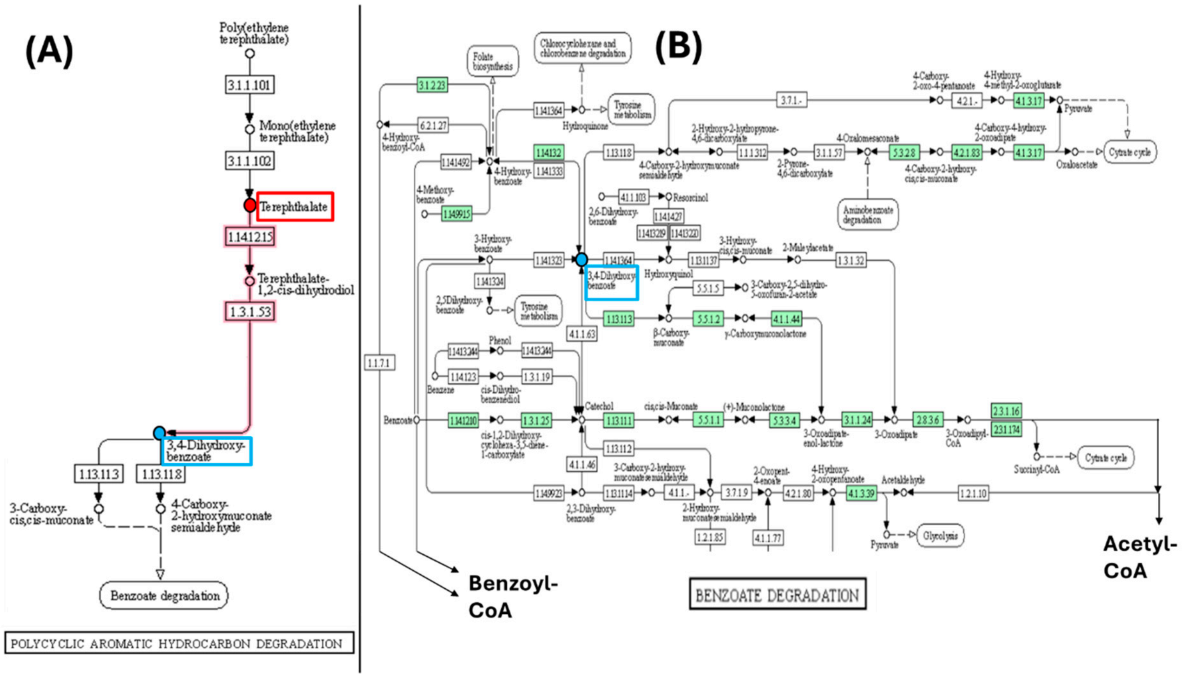

Supplemental Figure S1: Pathway of Consortium TPA metabolism.

(A) TPA metabolism begins at TPA highlighted in red and proceeds to 3,4 –dihydroxybenzoate. (B) 3,4-dihydroxybenzoate then proceeds through various pathways in the benzoate degradation pathway to the citrate cycle. The E.C numbers highlighted in green are enzymes that are important to our culture. The highlighted enzymes were identified using the pathway analysis function of MetaboAnalyst 6.0 using the 5 mM TPA cell-free extract LC-MS/MS data set. Pathway retrieved from KEGG [88,89].
